# Executive and non-executive functions in low birthweight/preterm adolescents with differing temporal patterns of inattention

**DOI:** 10.1371/journal.pone.0231648

**Published:** 2020-04-24

**Authors:** Marisa N. Spann, Anna Silberman, Judith Feldman, Steven J. Korzeniewski, J. Blake Turner, Agnes H. Whitaker

**Affiliations:** 1 Department of Psychiatry, Columbia University College of Physicians and Surgeons, New York, New York, United States of America; 2 Department of Psychiatry, New York State Psychiatric Institute, New York, New York, United States of America; 3 Department of Obstetrics & Gynecology, Wayne State University School of Medicine, Detroit, Michigan, United States of America; Universidad Nacional Autonoma de Mexico, MEXICO

## Abstract

**Objective:**

This study assesses whether low birthweight/preterm (LBW/PT) adolescents with persistent inattention (PIA) have neuropsychological deficits that distinguish them from adolescents with school age limited inattention (SAL) and those largely unaffected (UA).

**Method:**

Three latent classes (PIA, SAL, UA), derived from an earlier analysis of a LBW/PT birth cohort were compared on non-executive and executive functioning measures assessed at age 16.

**Results:**

The PIA class displayed the poorest performance on executive functioning, which was exaggerated in the context of lower IQ. The PIA and the SAL classes had poorer performance on non-executive functioning relative to the UA class. Both types of functioning mediated the relationship of class to school service use and grade retention.

**Conclusion:**

Neuropsychological impairment characterizes children and adolescents with inattention problems. Problems in executive functioning characterize the subset whose inattention persists through adolescence. Subsequent research can examine the potential for remediating these deficits to address academic and social problems.

## Introduction

Attention Deficit Hyperactivity Disorder (ADHD), a clinical diagnosis typically first made in childhood, is defined as the presence of a minimum number of inattentive (IA) and hyperactive-impulsive (HI behaviors, in varying proportions termed Predominantly HI, Predominantly IA and Combined presentations), accompanied by cross-situational impairment. ADHD is a public health concern: it affects 9% of school-age children [1, 2], and also is associated with suboptimal educational and occupational outcomes in adolescence and adulthood [3–6]. This is true even for the large majority (up to 70%) of children whose IA and HI symptoms fall below threshold for diagnosis by adolescence, and even more so for the substantial minority (30%) who still have an ADHD diagnosis, most commonly the Predominantly Inattentive presentation, as adolescents [7].

This heterogeneity in the presentation and trajectory of IA and HI behaviors has been a major challenge for the development of treatments for ADHD. Until now, much effort has focused on medications (primarily stimulants) and classroom and parenting management techniques that target ADHD behaviors–particularly HI behaviors. These have demonstrable short-term benefits [8–10] but questionable long-term benefits, assessed after two years [11, 12] in terms of educational and occupational outcomes. Because neuropsychological functions often mediate poor functional outcomes, and because these outcomes typically correspond to neuroimaging indicators of brain structure and function [13, 14], age-dependent neuropsychological assessment of cognitive functions may be a fruitful strategy to identify targets for intervention. On balance, the available evidence suggests that adolescents who have a history of childhood ADHD may share some early neuropsychological deficits that continue to impact learning [15–17], and that those who continue to have an ADHD diagnosis are additionally compromised in later emergent neuropsychological functions [4, 16–21] that otherwise compensate for or mitigate the impact of earlier deficits on educational outcomes [17, 19]. Previous studies of children and/or adolescents with persistent ADHD have primarily assessed two groups of neuropsychological functions [22, 23]. One group, often termed ‘non-executive’ (basic, not effortful), includes processing speed, memory, and sustained attention (see Table 1 for detailed description of these functions). These functions emerge early in development and appear to be dependent on subcortical structures [13]. The second group, often termed ‘executive’ (complex, effortful), does not emerge until later childhood or early adolescence. These functions continue to mature through early adulthood and appear to depend upon the maturation of cortical structures and cortical-subcortical connections [24]. Halperin and Schulz [22] suggested that the diagnosis of ADHD in childhood is associated with poor performance on tests of non-executive functions; for most of those with ADHD in childhood remittance of ADHD behaviors through adolescence is associated with adequate performance on tests of later developing executive functions. The literature thus far comparing neuropsychological profiles of adolescents whose ADHD persists versus remits is sparse, largely retrospective, and based primarily on clinical samples. Furthermore, the findings have been inconsistent [4, 15–20].

**Table 1:** Description of selected neuropsychological measures.

Cognitive Construct	Measure	Description
**Non-executive**		
Immediate (Immediate Memory) and long-term recognition (Long-term Memory)	Wechsler Memory Scale- Third Edition, Faces Subtest	Recognition of faces presented one at a time, immediately and following a delay.
Immediate/short-term (Immediate Memory) and long-term memory capacity (Long-term Memory; recall memory)	Wechsler Memory Scale- Third Edition, Logical Memory, Visual Reproduction, and Word Lists subtests	Immediate and delayed recall of stories, a list of words, and designs presented by the examiner.
Sustained attention (SA)	IVA, visual and auditory attention quotients	Participants respond to visual and auditory target and the percent of omission errors or failure to respond to a target item are calculated.
**Executive**		
Selective attention (visual search)	The Test of Everyday Attention for Children, Map Mission subtest	Participants search for target symbols among multiple distractors on a colored map, and the completion time is obtained.
Cognitive flexibility and inhibition	Trail Making Test, Part B	Participants must draw a line connecting consecutive numbers and letters, alternating between them, and the complete time is obtained.
Working memory	Wechsler Memory Scale–Third Edition, Letter Number Sequencing and Spatial Span subtests	Immediate recall and reorganization of letters, numbers, and spatial sequences.
Inhibition (inhibitory responses to non-targets)	Stroop Color Word Test, Interference Score	Participants must name the color of ink in which a word is printed, ignoring the word (e.g., The word red is printed in green ink).
Impulsivity	IVA, visual and auditory response control quotients	Participants respond to visual and auditory targets and the percent of commission errors or response to a non-target item are calculated.

Previous research suggests that deficits in neuropsychological functions are present for children with ADHD [15, 25] and children who are PT/LBW [26, 27] even when controlling for IQ. A few studies have considered IQ as a moderator of the association between ADHD symptoms and neuropsychological functions particularly executive functions [18, 28]. For example, Scott et al. [28] found that deficits associated with executive function in children with ADHD are largely limited to the lower IQ range.

The present study leverages information from a regional birth cohort of low birthweight/preterm infants assessed for individual ADHD behaviors at ages 6, 9 and 16. Previous research on this cohort has found the presentation and trajectory of their ADHD behaviors to be similar to those described for general population and term samples [29]. Latent class analysis of individual IA and HI symptoms assessed at the three ages in this cohort identified three groups: an unaffected class (UA) who had no more than mild HI and IA symptoms at age 6 and virtually none thereafter; a school age limited class (SAL) whose levels of HI and IA symptoms were both generally high at the age 9 assessment but substantially remitted by age 16; and a persistent inattentive class (PIA) characterized by the relatively high levels of both HI and IA symptoms at age 9 and, at age 16, similarly high levels of IA symptoms but much lower levels of HI symptoms. Educational outcomes in terms of special education service utilization and grade retention by age 16 were best in the unaffected class. These findings were not explained by differences in general cognitive or motor performance [29].

The analyses presented here examine non-executive and executive functions in the aforementioned regional birth cohort. Our objective is to: (1) describe the neuropsychological profiles of each of the three latent classes (UA, SAL and PIA) in terms of executive (e.g., selective attention, cognitive flexibility, working memory, inhibition, and impulsivity) and non-executive (e.g., processing speed, immediate and delayed memory, and sustained attention) functions; (2) determine the similarities and differences between the SAL and PIA groups in terms of non-executive and executive functions; (3) examine the extent to which IQ moderates the effect of inattention latent class profiles on executive functions; and (4) examine whether differences in performance on non-executive and executive across the three groups explain the elevated rates of educational difficulties in the PIA and SAL classes.

## Method

### Birth cohort and longitudinal assessment

The Neonatal Brain Hemorrhage Study (NBHS) birth cohort (n = 1105) included 90% of births <1500 grams and 85% of births <2000 grams in three New Jersey counties. The counties were demographically representative of the US as a whole [30, 31], from 1984 to 1987. The infants were screened for perinatal brain injury. After birth, a maternal interview and hospital chart abstraction were performed to obtain additional pre-, peri- and neo-natal data [30]. The cohort participated in neurodevelopmental evaluations at 2 [30, 31], 6 [32, 33], 9 [34] and 16 [35, 36] years of age. Neuropsychological data from the age 16 follow-up is presented here. This secondary analysis was approved by the New York State Psychiatric Institute Institutional Review Board and informed consent at the time of each study visit was obtained from the participants in person.

### Present sample

The study sample consists of the 387 participants for whom data from all three psychiatric assessment points (ages 6, 9, and 16) was available, and who did not have a major disability age 16 (IQ<55 or untestable or unable to walk without assistance). Earlier latent class analysis of ADHD criteria from the three assessments resulted in the PIA (n = 66), SAL (n = 150), and UA (n = 171) classes.

### Measures

#### ADHD

ADHD criteria were assessed using the Diagnostic Interview Schedule for Children (DISC) parent version [37]. The age 6 follow-up used the DISC-2.1 [38] based on DSM-III-R criteria [39]. The age 9 and 16 follow-ups used the DISC-3.0 and the DISC-IV, respectively, both based on DSM-IV [40].

#### Non-executive and executive functions

The non-executive functions measures include tests of sustained attention: Integrated Continuous Performance Test (IVA), visual and auditory Attention Quotients (AQ) and Hyperactivity Scale; visual memory: WMS-III Visual Immediate and Delayed Memory; and auditory memory: Wechsler Memory Scale–Third Edition (WMS-III), Auditory Immediate and Delayed Memory. The executive functions include measures of working memory: WMS-III Working Memory Index; cognitive flexibility: Trail Making Test- Part B (TMT-B); impulsivity: IVA, visual and auditory Response Control Quotients (RCQ); inhibition: the Stroop Color Word Interference Test (Stroop Interference); and selective attention: Test of Everyday Attention for Children (TEA-Ch), Map Mission subtest. Table 1 provides a description of these measures. The results from the subtests that encompass the IVA quotient (AQ and RCQ) or the WMS-III index (Working Memory) scores are presented in S2 Table.

#### Intellectual function

Intellectual function of LBW/PT status has the potential to be modify the relationship between ADHD symptoms patterns over time and our executive functions measures [28]. For this reason, we include in post-hoc analysis the Wechsler Abbreviated Scale of Intelligence [41] (WASI), a standardized assessment of general cognitive ability (IQ), administered at age 16.

#### Educational outcomes

We examine the extent to which differences in neuropsychological performance mediate the relationship between the latent classes and indicators of educational difficulties, including use of special education services and repeating a grade. Association of these outcomes with the latent classes was documented in earlier analyses [29].

### Statistical analysis

The main analyses for this study examined the associations of the latent classes with indicators of non-executive and executive functions at 16. The inferential statistics for these analyses involved omnibus tests of latent class differences in mean level (*F*) of the non-executive and executive measures. When warranted, post-hoc pairwise comparisons were conducted. The Type I error rate was set at 0.05 throughout. To account for multiple testing, we also report the Bonferroni correction for the analyses of neuropsychological measures (for 13 independent regression tests, *p* = 0.05/13 = 0.004).

Additional analyses recast the continuous measures of cognitive functioning into dichotomies representing impairment (defined as two or more standard deviations below the average score). The rates of impairment in each of the domains was compared across the three latent classes using chi-squared tests.

In line with previous research [28] demonstrating that the associations of inattention with neurocognitive deficits, including executive functions, are strongest in the context of lower IQ, we considered IQ (as measured by the WASI), as a moderator of latent class differences in executive functioning. These analyses involve regressions of executive function tests on latent class, IQ, and the interaction of IQ and latent class.

To assess the role of neuropsychological impairment in mediating the association of the latent classes to educational outcomes, a set of logistic regression models regressed lifetime use of school services and ever repeating a grade on the latent classes and, subsequently, the executive and non-executive functioning tests, first in separate blocks and then together.

The statistical models are adjusted for the child’s sex, gestational age at birth, and small for gestational age. The adjusted models are presented in the S1 and S3 Tables.

## Results

### Sample characteristics

Table 2 provides information regarding the maternal and birth demographics and information about the time of assessment and overall neurocognitive functioning during adolescence for the study sample. Only a small proportion of the mothers in the study had not completed high school (9.6%). Larger proportions were unmarried (14.7%) and on public assistance at the time of the child’s birth (14.5%). Children in the sample had an average birth weight of 1488 grams and gestational age of 31 weeks, and they had an equal number of males and females. Approximately 31% of the sample was small for gestational age.

**Table 2:** Descriptive characteristics for the study sample.

Variables	n/N	%
*Maternal*		
< High school education	37/387	9.6
Minority race	82/387	21.2
Marital status, unmarried	57/387	14.7
< 19 years of age at child’s birth	19/387	4.9
Public assistance at child’s birth	56/387	14.5
*Birth*		
Birth weight, grams, n, mean(SD)	387	1488.28(355.64)
Gestational age, completed weeks, n, mean(SD)	387	31.16(3.00)
Small for gestational age	118/387	30.5
Male sex	200/387	51.7
*Adolescent*		
Age at NP assessment, n, mean(SD)	369	15.17(0.41)
Years of education, n, mean(SD)	364	9.76(0.64)
WASI, SS, n, mean(SD)		
Full Scale IQ	381	99.38(14.37)
Verbal IQ	381	101.37(13.99)
Performance IQ	381	96.75(14.00)
Riley MPI total motor score, n, mean(SD)	382	3.54(3.15)
Not currently taking medication	268/387	69.3

Abbreviations: NP, neuropsychological; WASI, Wechsler Abbreviated Scale of Intelligence; IQ, intellectual quotient; SS, Standard Score; MPI, Motor Problems Inventory; SD, standard deviation.

### Non-executive and executive neuropsychological test scores by latent class

Table 3 shows the substantial differences in rates of both non-executive and executive functions across the three classes. In general, across the outcomes, the PIA class had lower (worse) performance relative to the SAL class, which, in turn, performed poorly relative to the UA class. The PIA class performed significantly worse relative to the SAL and UA classes across several measures of executive functions including inhibition (auditory), impulsivity, cognitive flexibility, and selective attention and non-executive functions including auditory memory (immediate and long-term). The PIA class, but not the SAL class performed significantly worse on a measure of executive function, inhibition (visual) and a measure of non-executive function, visual long-term memory compared to the UA class. As shown in S1 Table, the findings comparing the mean differences on non-executive and executive function tests among latent class groups remained essentially the same after controlling for child’s sex, gestational age at birth, and small for gestational age. As shown in S2 Table, the subtests that comprise the working memory, impulsivity, and sustained attention’ combined scores demonstrate a similar pattern of association as the combined score.

**Table 3:** Analyses of variance with neuropsychological measures organized by significant attention class differences.

		Persistent Inattentive n = 66		School Age Limited n = 150		Unaffected n = 171					Post-Hoc Analyses Mean Difference(*P* Value)		
Measures	Functions	Mean(n)	SD	Mean(n)	SD	Mean(n)	SD	F^(a)^	*P* Value^(b)^	η^2^	PIA vs SAL	SAL vs UA	PIA vs UA
**Non-executive**													
IVA Visual AQ, SS	Sustained Attention	81.38(63)	27.39	87.74(140)	23.91	97.79(166)	16.21	16.17	<0.0001	0.07	-6.36(0.15)	**-10.05(<0.0001)**	**-16.41(<0.0001)**
IVA Auditory AQ, SS	Sustained Attention	80.02(63)	23.40	85.10(140)	24.04	94.37(166)	16.88	13.12	<0.0001	0.06	-5.08(0.29)	**-9.28(0.001)**	**-14.36(<0.0001)**
IVA Hyperactivity, SS	Impulsivity	76.58(63)	31.44	81.98(140)	29.78	91.27(166)	24.78	7.84	<0.0001	0.04	-5.40(0.45)	**-9.29(0.02)**	**-14.68(0.002)**
WMS-III Auditory Immediate, SS	Immediate Memory	89.61(56)	15.61	97.31(134)	16.28	102.34(162)	13.79	15.42	<0.0001	0.08	**-7.70(0.006)**	**-5.03(0.02)**	**-12.73(<0.0001)**
WMS-III Auditory Delayed, SS	Long-term Memory	91.41(51)	19.89	99.35(131)	15.80	104.81(160)	12.75	15.92	<0.0001	0.08	**-7.94(0.007)**	**-5.46(0.01)**	**-13.39(<0.0001)**
WMS-III Visual Immediate, SS	Immediate Memory	93.93(54)	16.50	97.25(133)	15.78	100.83(162)	15.39	4.49	0.01	0.03	**—**	**—**	**—**
WMS-III Visual Delayed, SS	Long-term Memory	93.98(50)	18.50	99.86(131)	16.20	103.89(160)	16.46	7.13	0.001	0.04	-5.88(0.11)	-4.02(0.12)	**-9.91(0.001)**
**Executive**													
IVA Auditory RCQ, SS	Impulsivity	82.99(63)	24.82	91.58(140)	22.97	95.97(166)	18.82	8.34	<0.0001	0.21	**-8.58(0.03)**	-4.39(0.21)	**-12.98(<0.0001)**
IVA Visual RCQ, SS	Impulsivity	84.38(63)	25.81	89.64(140)	25.30	93.84(166)	19.97	4.01	0.02	0.15	—	—	**—**
Stroop Interference, SS	Inhibition	87.08(64)	7.85	90.74(136)	6.93	92.23(162)	6.13	13.32	<0.0001	0.26	**-3.66(0.002)**	-1.49(0.17)	**-5.15(<0.0001)**
TEA-Ch Map Mission, ss	Selective Attention	8.80(56)	3.71	10.18(130)	2.85	11.15(155)	2.84	13.19	<0.0001	0.27	**-1.37(0.02)**	**-0.98(0.02)**	**-2.35(<0.0001)**
TMT–B, zs	Cognitive Flexibility	-7.23(56)	6.17	-4.26(137)	3.71	-3.33(160)	3.22	19.68	<0.0001	0.32	**-2.97(<0.0001)**	-0.93(0.14)	**-3.90(<0.0001)**
WMS-III Working Memory, SS	Working Memory	91.65(51)	16.23	94.98(132)	15.96	103.28(160)	12.03	18.74	<0.0001	0.09	-3.34(0.37)	**-8.29(<0.0001)**	**-11.63(<0.0001)**

^**(a)**^ Two degrees of freedom

^**(b)**^ P values are exact 2-sided.

Statistically significant values of p<0.05 are shown in bold; p<0.004 meets significance based on Bonferroni correction

Abbreviations for Measures: WMS–III, Wechsler Memory Scale–Third Edition; IVA, Integrated Visual and Auditory Continuous Performance Test; RCQ, Response Control Quotient; TEA-Ch, Test of Everyday Attention for Children, TMT–B, Trail Making Test, Part B; Stroop Interference, Stroop Color and Word Test, Interference Score; SS, Standard Score; ss, Scaled Score; zs, Z Score; SD, standard deviation; UA, Unaffected; SAL, School Age Limited; PIA, Persistent Inattentive.

### Neuropsychological impairment by latent class

In addition to mean differences by latent classes on the neuropsychological test scores, we also examined differences in rates of neuropsychological impairment (defined as two standards deviations or greater from the mean) across the classes (Table 4). The PIA class generally had higher rates of impaired performance relative both to the SAL and UA classes. Relative to the UA group the differences were consistent across all of the classes and were mostly statistically significant. Relative to the SAL class there were significant differences for three of the four executive function tests including impulsivity, selective attention, and cognitive flexibility; there were also substantial and marginally significant differences on the auditory memory tests. In contrast, the SAL class relative to the UA class only had higher rates of impairment on two non-executive function measures, auditory immediate and delayed memory. As shown in S3 Table, the findings comparing the latent classes on rates of non-executive and executive functional impairment remained essentially the same after controlling for the birth risk factors noted above.

**Table 4:** Chi-square of below average performance on neuropsychological measures and attention classification.

Measures	Functions	Persistent Inattentive n = 66		School Age Limited n = 150		Unaffected n = 171		*X*^2^^(a)^	*P* Value^(b)^	Post-Hoc Analyses					
										PIA vs SAL		SAL vs UA		PIA vs UA	
		n/N	%	n/N	%	n/N	%			% diff	P value	% diff	P value	% diff	P value
**Nonexecutive**															
WMS–III Auditory Immediate	Immediate Memory	20/56	35.7	29/134	21.6	13/162	8.0	24.41	<0.0001	14.1	0.06	13.6	**0.007**	27.7	**<0.0001**
WMS–III Auditory Delayed	Long-term Memory	17/51	33.3	25/131	19.1	14/160	8.8	18.21	<0.0001	14.2	0.06	10.3	0.054	24.5	**<0.0001**
WMS-III Visual Immediate	Immediate Memory	16/54	29.6	31/133	23.3	27/162	16.7	4.64	0.098	6.3	0.63	6.6	0.38	12.9	0.13
WMS–III Visual Delayed	Long-term Memory	15/50	30.0	25/131	19.1	18/160	11.3	10.14	0.006	10.9	0.21	7.8	0.20	18.7	**0.008**
**Executive**															
IVA Auditory RCQ, SS	Impulsivity	18/63	28.6	19/140	13.6	14/166	8.4	15.56	<0.0001	15	**0.02**	5.2	0.42	20.2	**<0.0001**
IVA Visual RCQ, SS	Impulsivity	15/63	23.8	23/140	16.4	19/166	11.4	5.51	0.06	7.4	0.40	5	0.48	12.4	0.07
Stroop Interference, SS	Inhibition	0/64	0.0	0/136	0.0	0/162	0.0	---	---	0	---	0	---	0	---
TEA-Ch Map Mission, ss	Selective Attention	7/56	12.5	1/130	0.8	2/155	1.3	21.61	<0.0001	11.7	**<0.0001**	0.5	0.97	11.2	**<0.0001**
TMT–B, zs	Cognitive Flexibility	51/56	91.1	100/137	73.0	101/160	63.1	16.14	<0.0001	18.1	**0.04**	9.9	0.16	28	**<0.0001**

^**(a)**^ Two degrees of freedom.

^**(b)**^ *P* values are exact 2-sided.

Statistically significant values of p<0.05 are shown in bold; p<0.004 meets significance based on Bonferroni correction

Note: All data presented is comparing attention classes based on performance on the cognitive measure of > 2 standard deviations below the mean.

Abbreviations for Measures: WMS–III, Wechsler Memory Scale–Third Edition; IVA, Integrated Visual and Auditory Continuous Performant Test; RCQ, Response Control Quotient; TEA-Ch, Test of Everyday Attention for Children, TMT–B, Trail Making Test, Part B; Stroop Interference, Stroop Color and Word Test, Interference Score; SS, Standard Score; ss, Scaled Score; zs, Z Score; SD, standard deviation; UA, Unaffected; SAL, School Age Limited; PIA, Persistent Inattentive.

### IQ as a moderator of the effects of inattention on executive functioning

**Fig 1:**
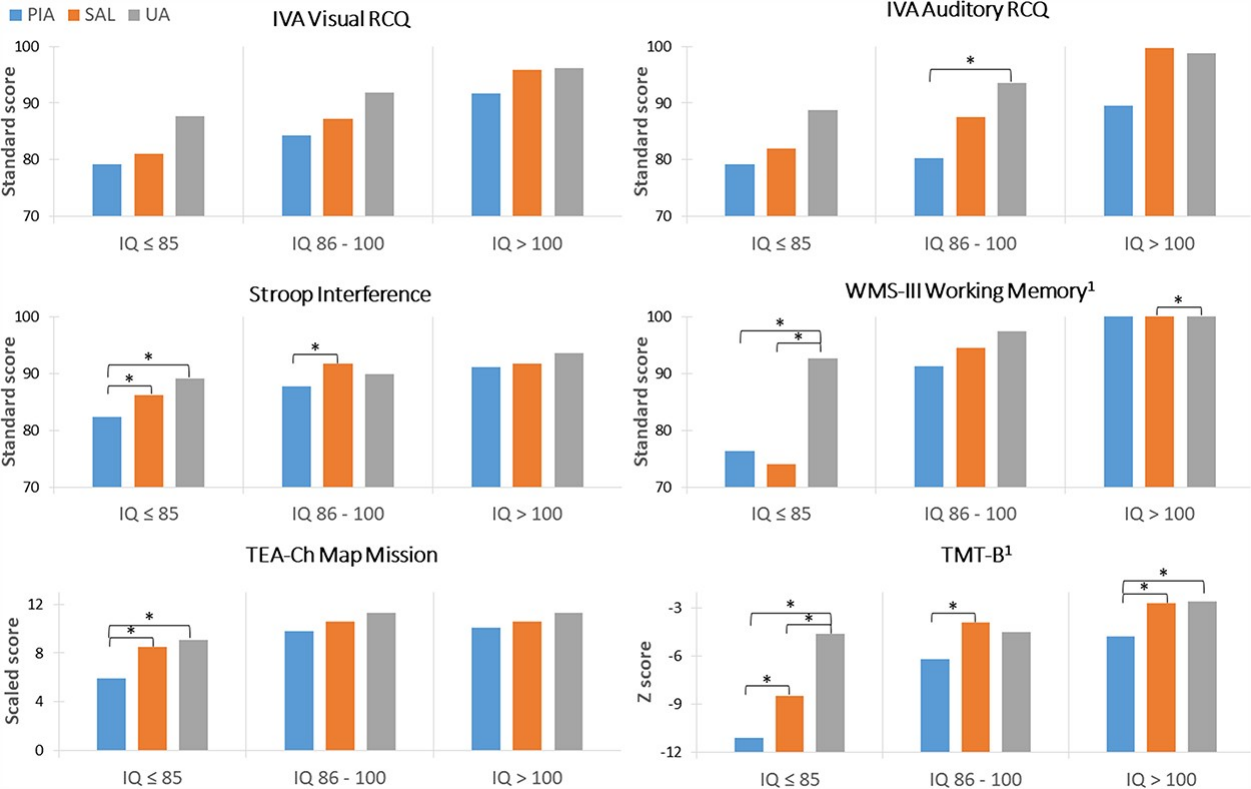
IQ as a moderator of the effects of inattention on executive functioning. We show the mean differences of the PIA and SAL classes relative to the UA class, estimated at three different levels of IQ (≤ 85, 86–100, and >100). For the TMT-B and the WMS-III Working Memory measures, lower IQ range greatly exacerbated the effects of being in the PIA and the SAL classes, relative to the effects within the other two IQ categories. These interactions were statistically significant. ^1^denotes a significant interaction (p<0.05) between IQ and the executive function measure. *denotes a significant difference (p<0.05) between two latent classes on the neuropsychological measure.

Fig 1 displays the mean differences of the PIA and SAL classes relative to the UA class, estimated at three different levels of IQ. Among those with average to above average IQ scores, further increases in IQ did not influence the relationship between latent class and the study outcomes. Thus, adolescents with higher IQ scores (> 100) were collapsed into a single category and compared to those whose IQ was < = 85 and those whose IQ scores were > 85 and < = 100.

For the TMT-B z-score and the WMS-III Working Memory tests, lower IQ greatly exacerbated the association between the PIA and the SAL classes and study outcomes, relative to the relationships within the other two IQ categories (Fig 1). These interactions were statistically significant. For the Stroop Interference and TEA-Ch Map Mission tests, a similar pattern emerged. While the interaction terms themselves were not significant, the gaps between the PIA and UA groups on these outcomes are large and statistically significant within the lower IQ group, while the same contrasts at higher IQ level are much smaller and non-significant. IQ-level does not seem to modify the effects of being in the PIA and SAL classes on the ARCQ and VRCQ tests.

### Neuropsychological test scores as mediators of educational difficulties for the IPA and SAL classes

Table 5 presents the results of analysis in which neuropsychological test scores are considered as potential mediators of the elevated rates of academic difficulties (operationally-defined as use of school services and repeating a grade) in PIA and SAL youth. Non-executive functioning mediates about 28% and 21% of the elevated rates of school service use in the PIA and SAL groups, respectively. Executive functioning appears to play a somewhat greater role, particularly for the PIA group. The executive functioning tests mediate more than half of the PIA elevation and about 30% of the SAL elevation. Together, both types of test mediate 58% of the elevation of PIA and 33% of the SAL elevation.

**Table 5:** Neuropsychological functioning domains as mediators of the relation of temporal patterns of inattention to educational difficulties.

	Odds Ratio	95% CI	% change log(OR)	Odds Ratio	95% CI	% change log(OR)
**A. Ever used school services**						
Total Effect	4.65	2.11–10.3		3.22	1.74–5.95	
Adjusting for:						
Executive functioning	2.03	0.80–5.14	54.1	2.26	1.12–4.56	30.2
Non-executive functioning	3.02	1.27–7.21	28.1	2.52	1.31–4.87	20.7
Both	1.91	0.73–4.99	58.0	2.19	1.07–4.50	32.8
**B. Ever repeated a grade**						
Total Effect	2.86	0.94–8.73		3.55	1.52–8.28	
Adjusting for:						
Executive functioning	1.24	0.34–4.54	79.5	3.01	1.18–7.71	12.9
Non-executive functioning	1.91	0.58–6.33	38.4	3.08	1.26–7.52	11.4
Both	1.15	0.30–4.40	87.1	3.09	1.19–8.02	10.9

The picture is somewhat different for repeating a grade. For this outcome the mediating role of the neuropsychiatric tests is substantial for the PIA group–particularly with respect to the executive functioning tests. Nearly 80% of the elevation for the PIA group is mediated by executive functioning, all of the tests together mediate 87%. In contrast, the mediation effects for the SAL group are quite small for both types of test. As shown in S3 Table, the findings are largely similar with adjustments for child sex, gestational age at birth, and small for gestational age. The results demonstrate even greater influence on the executive functioning domain.

## Discussion

The analyses in this paper employed one of few extant datasets having longitudinal data on DSM ADHD criteria, as well as clinical characteristics and functional outcomes, in a prospective LBW/PT birth cohort [29, 30]. We compared three latent classes, derived from temporal patterns of ADHD symptoms and behaviors, in terms of their neuropsychological test performance at age 16. The persistently inattentive (PIA) class significantly differed from the school-age limited (SAL) and unaffected (UA) classes on a subset of executive function tests including, inhibition, impulsivity, and cognitive flexibility and on two non-executive function tests, auditory immediate and delayed memory. This difference was particularly dramatic in the context of lower IQ on the executive function tests. In contrast, the SAL class differed from the UA class primarily in the nonexecutive function tests. The findings were similar, with a few exceptions, when the extreme end of performance (less that than two standard deviations below the normed average) on these tests was compared. Performance on executive and non-executive measures mediate elevated rates of school service in both the PIA and SAL classes, and elevated rates of repeating a grade only for the PIA class. Executive function measures were primarily responsible for the mediation. Overall, these findings suggest that children born LBW/PT whose inattentive behaviors persist into later adolescence exhibit pronounced cognitive disinhibition, impulsivity, and flexibility deficits. These executive functioning deficits, in turn, seem to impair educational attainment. Our finding of poorer performance of the PIA class compared to the UA class across both executive and non-executive function tests aligns with prior studies [4, 15]. Unique to our analyses is the contrast in neuropsychological functioning between groups whose school-age inattention behaviors persisted well into adolescence and those whose inattention remitted. These two classes (PIA versus SAL) differ largely in terms of executive function. Non-executive function deficits remain for the SAL class in spite of the largely complete remission of their inattention behaviors and their ADHD behaviors in general. This contrasts with earlier studies conducted in adolescents not selected for low birthweight or prematurity that found no differences between groups with remitting versus persisting ADHD diagnoses [4, 17, 20]. The specificity of the cognitive functions that differentiate the two classes may enhance the precision of behavioral and neurofeedback interventions that can specifically target and thereby improve these central executive functions.

Consistent with a few other studies [18, 28], we considered IQ as a potential modifier of the association between ADHD symptoms and neuropsychological functions particularly executive functions. The extremely low gestational age newborn (ELGAN) study found that children who screened positive for ADHD had increased risk of impaired performance in working memory and inhibition in the context of lower IQ (<70 and <85). In our PT/LBW cohort across the three latent classes, we also found a similar interaction between lower IQ (< 85) and the executive functions working memory and impulsivity, but not inhibition and selective attention. Lower IQ clearly does not account for all of the differences in executive functions that we identified across the three latent classes, however it is an important cognitive construct to consider in these types of studies. For example, studies that focus on developing cognitive remediation programs of executive functions should take into account the IQ level of the participants to ensure there are modifiable activities within the program that target the same executive function.

Our findings are broadly consistent with the Halperin and Schulz neurodevelopmental model of ADHD recovery [22]. Both latent classes with school age inattention behaviors generally performed poorly on non-executive function tests compared to the UA class. These non-executive processes rely on subcortical brain regions, such as the basal ganglia, that develop in early childhood. It is plausible then that such deficits are less amenable to improvements later in adolescence [22, 42].

The fact that the SAL class has far fewer deficits in executive functions—which rely on late developing cortical brain regions such as the prefrontal cortex–could be in-line with three alternative perspectives. First, remittance of inattention problems could support the improvement of these executive function abilities in adolescence. Second, the emergence of executive function abilities could enable adolescents to overcome their inattention behaviors. Finally, the rapid development of the prefrontal cortex that typical occurs in adolescence [24] enhances both executive functioning and improves attention. This last interpretation presumes a deficit in late frontal lobe development in youth with long-term persistent inattention problems through adolescence [22]. A longitudinal study of a LBW/PT cohort found that those with persistent attentive/hyperactive behaviors in childhood (relative to term children) demonstrated decreased volume in the dorsal prefrontal and orbitofrontal cortices as neonates [43]. These brain regions were not significantly different from LBW/PT with no or transient attentive/hyperactive behaviors; however sensorimotor brain regions differed. Unfortunately, because the no or transient attentive/hyperactive group was combined, we cannot be certain whether one or both differed from the persistent group. Thus, persistence of these executive functioning deficits in the PIA vs. SAL class in the context of LBW/PT may be associated with differential development of some brain regions however further studies are necessary for this to be determined. Regardless of the causal pathway, the two latent classes clearly have neuropsychological and behavioral differences that distinguish them. Future studies that include serial magnetic resonance imaging (MRI), neuropsychological assessments, and symptom reports will have the capacity to unravel how these three domains of assessment relate.

We further examined executive and non-executive functioning as potential mediators of elevated rates of educational problems in the PIA and SAL classes. Neuropsychological functioning, in particular executive functioning, is a stronger mediator of the elevated rates educational problems in the PIA class. The two domains of functioning have similar mediation effects for the SAL class and, in combination, are not as strong as the mediation for PIA. Indeed, elevated rate of repeating a grade in the SAL class is largely unchanged when functioning is included in the model. This may be because members of the SAL class are likely to have repeated a grade earlier in childhood during the period when they had high levels of inattention. If this were the case, their (likely improved) neuropsychological functioning are age 16 would be less strongly associated with that outcome.

This study advances our understanding of the neuropsychological profiles of children with differing trajectories of inattention problems through childhood and adolescence. Nonetheless, it should be noted that the classes of subjects identified in this study may have been different had children with full-term gestation and typical birth weight been included. Further, the associations of ADHD symptom patterns with cognitive functioning measures might not be generalizable to the general population. It should also be noted that data on other deficits that have been associated with inattention problems, such as deficits in planning [15], were not assessed in this cohort. Finally, only a subset of the entire cohort completed three diagnostic assessments, which limited the current study sample size. However, the current subsample is similar to the entire cohort in their to prenatal and birth characteristics [29].

## Conclusion

The current study expands the existing literature aiming to differentiate children with varying durations of inattention problems throughout adolescence. Using a unique LBW/PT sample, enriched for the occurrence of these problems [29, 44], we were able to demonstrate differences in executive and non-executive functions between the PIA and SAL classes in the areas of inhibition, impulsivity, and cognitive flexibility. These deficits mediate rates of reported educational difficulties primarily children with inattention problems persist through adolescence. Given that adolescents with ADHD and those born prematurely are more likely to be limited in their educational attainment and to have difficulties with employment [3, 42, 45], and that executive functions appear to mediate these deficits (at least in the case of educational problems), improvement of the specific executive functions through intervention is an important consideration both during adolescence and the transition into emerging adulthood. ADHD in early adolescence and adulthood is also associated with various negative social outcomes, including an increased risk of imprisonment and violent offenses, drug use, and suicide attempts [46–48]. Executive-functioning based interventions for those with ADHD may help reduce the risk of these adverse outcomes.

## Supporting information

S1 Table Unadjusted and adjusted models for analyses of variance with neuropsychological measures, attention classification, and birth risk factors. (DOCX)

S2 Table Analyses of variance with neuropsychological measures including subtests organized by significant attention class differences. (DOCX)

S3 Table Unadjusted and adjusted models for chi-square of below average performance on neuropsychological measures, attention classification, and birth risk factors. (DOCX)
