# Supplementary material for: Executive and non-executive functions in low birthweight/preterm adolescents with differing temporal patterns of inattention
Source: PLoS One. 2020 Apr 24;15(4):e0231648. doi: 10.1371/journal.pone.0231648 (PMC7182186; doi:10.1371/journal.pone.0231648)
Supplement: S1 Table — (DOCX) [file pone.0231648.s001.docx]

Table S1. Unadjusted and Adjusted Models for Analyses of Variance with Neuropsychological Measures, Attention Classification, and Birth Risk Factors

|  |  | **Unadjusted** | | | |  | **Adjusted** | | | |
| --- | --- | --- | --- | --- | --- | --- | --- | --- | --- | --- |
| **Measures** | **Functions** | **Mean^2^** | **R^2^** | **Adjusted R^2^** | **F^(a)^*(P* Value)^(b)^** |  | **Mean^2^** | **R^2^** | **Adjusted R^2^** | **F^(a)^*(P* Value)^(b)^** |
| **Nonexecutive** |  |  |  |  |  |  |  |  |  |  |
| IVA Visual AQ | Sustained Attention | 7483.51 | 0.08 | 0.08 | 16.17(<0.0001) |  | 5443.31 | 0.13 | 0.11 | 12.25(<0.0001) |
| IVA Auditory AQ | Sustained Attention | 5939.88 | 0.07 | 0.06 | 13.12(<0.0001) |  | 5099.88 | 0.09 | 0.08 | 11.47(<0.0001) |
| IVA Hyperactivity Scale | Impulsivity | 6119.77 | 0.04 | 0.04 | 7.84(<0.0001) |  | 5474.53 | 0.05 | 0.04 | 7.02(0.001) |
| WMS – III Auditory Immediate | Immediate Memory | 3502.09 | 0.08 | 0.08 | 15.42(<0.0001) |  | 3009.83 | 0.12 | 0.11 | 14.16(<0.0001) |
| WMS – III Auditory Delayed | Long-term Memory | 3667.93 | 0.09 | 0.08 | 15.92(<0.0001) |  | 3026.21 | 0.12 | 0.11 | 13,90(<0.0001) |
| WMS-III Visual Immediate | Immediate Memory | 1108.31 | 0.03 | 0.02 | 4.49(0.01) |  | 739.26 | 0.08 | 0.07 | 3.52(0.04) |
| WMS – III Visual Delayed | Long-term Memory | 1981.64 | 0.04 | 0.04 | 7.13(0.001) |  | 1328.42 | 0.10 | 0.09 | 5.01(0.007) |
| **Executive** |  |  |  |  |  |  |  |  |  |  |
| IVA Visual RCQ, SS | Impulsivity | 2150.44 | 0.02 | 0.02 | 4.01(0.02) |  | 1979.18 | 0.03 | 0.02 | 3.71(0.03) |
| IVA Auditory RCQ, SS | Impulsivity | 3873.78 | 0.04 | 0.04 | 8.34(<0.0001) |  | 2530.30 | 0.08 | 0.07 | 5.64(0.004) |
| Stroop Interference, SS | Inhibition | 608.49 | 0.07 | 0.06 | 13.32(<0.0001) |  | 535.63 | 0.10 | 0.09 | 11.97(<0.0001) |
| TEA-Ch Map Mission, SS | Selective Attention | 118.75 | 0.07 | 0.07 | 13.19(<0.0001) |  | 110.93 | 0.09 | 0.07 | 12.28(<0.0001) |
| TMT – B, zs | Cognitive Flexibility | 315.34 | 0.10 | 0.10 | 19.68(<0.0001) |  | 272.61 | 0.11 | 0.10 | 17.14(<0.0001) |
| WMS-III Working Memory | Working Memory | 3833.55 | 0.10 | 0.09 | 18.74(<0.0001) |  | 3003.55 | 0.15 | 0.13 | 15.24(<0.0001) |

**^(a)^** Two degrees of freedom

**^(b)^** *P* values are exact 2-sided.

**^(c)^** Adjusted for the child’s sex, gestational age at birth, and small for gestational.

Statistically significant values of p<0.05 are shown in bold; p<0.004 meets significance based on Bonferroni correction

Abbreviations: WMS – III, Wechsler Memory Scale – Third Edition; IVA, Integrated Visual and Auditory Continuous Performant Test; AQ, Attention Quotient; RCQ, Response Control Quotient; TEA-Ch, Test of Everyday Attention for Children, TMT – B, Trail Making Test, Part B; Stroop Interference, Stroop Color and Word Test, Interference Score; SS, Standard Score; ss, Scaled Score; zs, Z Score; SD, standard deviation; UA, Unaffected; SAL, School Age Limited; PIA, Persistent Inattentive.
