# Supplementary material for: Executive and non-executive functions in low birthweight/preterm adolescents with differing temporal patterns of inattention
Source: PLoS One. 2020 Apr 24;15(4):e0231648. doi: 10.1371/journal.pone.0231648 (PMC7182186; doi:10.1371/journal.pone.0231648)
Supplement: S2 Table — (DOCX) [file pone.0231648.s002.docx]

Table S2. Analyses of Variance with Neuropsychological Measures including subtests organized by Significant Attention Class Differences

|  |  | **Persistent Inattentive**  **n=66** | | **School Age Limited**  **n=150** | | **Unaffected**  **n=171** | |  |  |  | **Post-Hoc Analyses**  **Mean Difference(*P* Value)** | | |
| --- | --- | --- | --- | --- | --- | --- | --- | --- | --- | --- | --- | --- | --- |
| **Measures** | **Functions** | **Mean(n)** | **SD** | **Mean(n)** | **SD** | **Mean(n)** | **SD** | **F^(a)^** | ***P* Value^(b)^** | **η^2^** | **PIA vs SAL** | **SAL vs UA** | **PIA vs UA** |
| **Non-executive** |  |  |  |  |  |  |  |  |  |  |  |  |  |
| IVA Visual AQ, SS | Sustained Attention | 81.38(63) | 27.39 | 87.74(140) | 23.91 | 97.79(166) | 16.21 | 16.17 | <0.0001 | 0.07 | -6.36(0.15) | **-10.05(<0.0001)** | **-16.41(<0.0001)** |
| Visual Speed, SS | Processing Speed | 92.87(63) | 22.14 | 94.39(140) | 19.06 | 101.60(166) | 15.38 | 8.38 | <0.0001 | 0.04 | -1.53(0.86) | **-7.21(0.003)** | **-8.74(0.005)** |
| Vigilance Visual Scale, SS | Sustained Attention | 78.29(63) | 33.94 | 86.93(140) | 27.40 | 97.47(166) | 16.28 | 16.02 | <0.001 | 0.07 | -8.63(0.07) | **-10.55(0.001)** | **-19.18(<0.001)** |
| Focus Visual Scale, SS | Sustained Attention | 86.78(63) | 20.14 | 90.82(140) | 20.55 | 95.94(166) | 19.34 | 5.56 | 0.004 | 0.03 | --- | --- | --- |
| IVA Auditory AQ, SS | Sustained Attention | 80.02(63) | 23.40 | 85.10(140) | 24.04 | 94.37(166) | 16.88 | 13.12 | <0.0001 | 0.06 | -5.08(0.29) | **-9.28(0.001)** | **-14.36(<0.0001)** |
| Auditory Speed, SS | Processing Speed | 86.53(63) | 17.88 | 83.76(140) | 18.31 | 88.82(166) | 17.45 | 3.04 | 0.049 | 0.02 | --- | --- |  |
| Vigilance Auditory Scale, SS | Sustained Attention | 77.64(63) | 32.77 | 85.67(140) | 26.70 | 94.52(166) | 18.34 | 12.04 | <0.001 | 0.06 | -8.03(0.10) | **-8.85(0.008)** | **-16.88(<0.001)** |
| Focus Auditory Scale, SS | Sustained Attention | 95.30(63) | 18.51 | 100.48(140) | 18.38 | 105.36(166) | 14.21 | 9.02 | <0.001 | 0.04 | -5.18(0.12) | **-4.88(0.03)** | **-10.06(<0.001)** |
| IVA Hyperactivity, SS | Impulsivity | 76.58(63) | 31.44 | 81.98(140) | 29.78 | 91.27(166) | 24.78 | 7.84 | <0.0001 | 0.04 | -5.40(0.45) | **-9.29(0.02)** | **-14.68(0.002)** |
| WMS-III Auditory Immediate, SS | Immediate Memory | 89.61(56) | 15.61 | 97.31(134) | 16.28 | 102.34(162) | 13.79 | 15.42 | <0.0001 | 0.08 | **-7.70(0.006)** | **-5.03(0.02)** | **-12.73(<0.0001)** |
| WMS-III Auditory Delayed, SS | Long-term Memory | 91.41(51) | 19.89 | 99.35(131) | 15.80 | 104.81(160) | 12.75 | 15.92 | <0.0001 | 0.08 | **-7.94(0.007)** | **-5.46(0.01)** | **-13.39(<0.0001)** |
| WMS-III Visual Immediate, SS | Immediate Memory | 93.93(54) | 16.50 | 97.25(133) | 15.78 | 100.83(162) | 15.39 | 4.49 | 0.01 | 0.03 | --- | --- | --- |
| WMS-III Visual Delayed, SS | Long-term Memory | 93.98(50) | 18.50 | 99.86(131) | 16.20 | 103.89(160) | 16.46 | 7.13 | 0.001 | 0.04 | -5.88(0.11) | -4.02(0.12) | **-9.91(0.001)** |
| **Executive** |  |  |  |  |  |  |  |  |  |  |  |  |  |
| IVA Auditory RCQ, SS | Impulsivity | 82.99(63) | 24.82 | 91.58(140) | 22.97 | 95.97(166) | 18.82 | 8.34 | <0.0001 | 0.21 | **-8.58(0.03)** | -4.39(0.21) | **-12.98(<0.0001)** |
| Stamina Auditory Scale, SS | Impulsivity | 98.59(63) | 17.61 | 99.96(140) | 19.91 | 100.08(166) | 15.62 | 0.17 | 0.84 | 0.001 | --- | --- | --- |
| Consistency Auditory Scale, SS | Impulsivity | 93.79(63) | 15.56 | 96.32(140) | 18.92 | 102.37(166) | 14.81 | 8.25 | <0.001 | 0.04 | -2.52(0.94) | **-6.05(0.007)** | **-8.59(0.002)** |
| Prudence Auditory Scale, SS | Impulsivity | 72.30(63) | 36.03 | 86.24(140) | 29.36 | 89.18(166) | 28.21 | 7.32 | 0.001 | 0.04 | **-13.94(0.007)** | -2.94(1.00) | **-16.88(0.001)** |
| IVA Visual RCQ, SS | Impulsivity | 84.38(63) | 25.81 | 89.64(140) | 25.30 | 93.84(166) | 19.97 | 4.01 | 0.02 | 0.15 | --- | --- | --- |
| Stamina Visual Scale, SS | Impulsivity | 100.61(63) | 19.88 | 97.72(140) | 15.69 | 98.11(166) | 13.07 | 0.81 | 0.44 | 0.009 | --- | --- | --- |
| Consistency Visual Scale, SS | Impulsivity | 91.50(63) | 16.69 | 94.17(140) | 20.51 | 101.57(166) | 15.15 | 10.46 | <0.001 | 0.05 | -2.67(0.95) | **-7.40(0.001)** | **-10.07(0.001)** |
| Prudence Visual Scale, SS | Impulsivity | 77.68(63) | 31.97 | 88.02(140) | 28.34 | 88.50(166) | 25.69 | 3.80 | 0.02 | 0.02 | --- | --- | --- |
| Stroop Interference, SS | Inhibition | 87.08(64) | 7.85 | 90.74(136) | 6.93 | 92.23(162) | 6.13 | 13.32 | <0.0001 | 0.26 | **-3.66(0.002)** | -1.49(0.17) | **-5.15(<0.0001)** |
| TEA-Ch Map Mission, ss | Selective Attention | 8.80(56) | 3.71 | 10.18(130) | 2.85 | 11.15(155) | 2.84 | 13.19 | <0.0001 | 0.27 | **-1.37(0.02)** | **-0.98(0.02)** | **-2.35(<0.0001)** |
| TMT – B, zs | Cognitive Flexibility | -7.23(56) | 6.17 | -4.26(137) | 3.71 | -3.33(160) | 3.22 | 19.68 | <0.0001 | 0.32 | **-2.97(<0.0001)** | -0.93(0.14) | **-3.90(<0.0001)** |
| WMS-III Working Memory, SS | Working Memory | 91.65(51) | 16.23 | 94.98(132) | 15.96 | 103.28(160) | 12.03 | 18.74 | <0.0001 | 0.09 | -3.34(0.37) | **-8.29(<0.0001)** | **-11.63(<0.0001)** |
| LN Sequencing, ss | Working Memory | 8.42(52) | 3.33 | 9.13(132) | 3.10 | 10.67(161) | 2.60 | 16.32 | <0.0001 | 0.30 | -0.71(0.34) | **-1.54(<0.0001)** | **-2.24(<0.0001)** |
| Spatial Span, ss | Working Memory | 8.51(51) | 3.16 | 9.11(132) | 3.15 | 10.56(160) | 2.31 | 15.28 | <0.0001 | 0.29 | -0.60(0.43) | **-1.46(<0.0001)** | **-2.05(<0.0001)** |

**^(a)^** Two degrees of freedom

**^(b)^** P values are exact 2-sided.

Statistically significant values of p<0.05 are shown in bold; 0.05/27=p<0.002 meets significance based on Bonferroni correction

Abbreviations for Measures: WMS – III, Wechsler Memory Scale – Third Edition; IVA, Integrated Visual and Auditory Continuous Performant Test; RCQ, Response Control Quotient; TEA-Ch, Test of Everyday Attention for Children, TMT – B, Trail Making Test, Part B; Stroop Interference, Stroop Color and Word Test, Interference Score; SS, Standard Score; ss, Scaled Score; zs, Z Score; SD, standard deviation; UA, Unaffected; SAL, School Age Limited; PIA, Persistent Inattentive.
