# Supplementary material for: Executive and non-executive functions in low birthweight/preterm adolescents with differing temporal patterns of inattention
Source: PLoS One. 2020 Apr 24;15(4):e0231648. doi: 10.1371/journal.pone.0231648 (PMC7182186; doi:10.1371/journal.pone.0231648)
Supplement: S3 Table — (DOCX) [file pone.0231648.s003.docx]

Table S3. Unadjusted and Adjusted Models for Chi-square of Below Average Performance on Neuropsychological Measures, Attention Classification, and Birth Risk Factors.

|  |  | **Persistent Inattentive**  **n=66** | | **School Age Limited**  **n=150** | | **Unaffected**  **n=171** | | ***X*^2(a)^( *P* Value^(b^)** | |
| --- | --- | --- | --- | --- | --- | --- | --- | --- | --- |
| **Measures** | **Functions** | **n/N** | **%** | **n/N** | **%** | **n/N** | **%** | **Unadj.** | **Adj.^(c)^** |
| **Nonexecutive** |  |  |  |  |  |  |  |  |  |
| WMS – III Auditory Immediate | Immediate Memory | 20/56 | 35.7 | 29/134 | 21.6 | 13/162 | 8.0 | 24.41(<0.0001) | 18.85(<0.0001) |
| WMS – III Auditory Delayed | Long-term Memory | 17/51 | 33.3 | 25/131 | 19.1 | 14/160 | 8.8 | 18.21(<0.0001) | 15.82(<0.0001) |
| WMS-III Visual Immediate | Immediate Memory | 16/54 | 29.6 | 31/133 | 23.3 | 27/162 | 16.7 | 4.64(0.098) | 2.55(0.28) |
| WMS – III Visual Delayed | Long-term Memory | 15/50 | 30.0 | 25/131 | 19.1 | 18/160 | 11.3 | 10.14(0.006) | 5.26(0.07) |
| **Executive** |  |  |  |  |  |  |  |  |  |
| IVA Visual RCQ, SS | Impulsivity | 15/63 | 23.8 | 23/140 | 16.4 | 19/166 | 11.4 | 5.51(0.06) | 8.74(0.01) |
| IVA Auditory RCQ, SS | Impulsivity | 18/63 | 28.6 | 19/140 | 13.6 | 14/166 | 8.4 | 15.56(<0.0001) | 10.17(0.006) |
| Stroop Interference, SS | Inhibition | 0/64 | 0.0 | 0/136 | 0.0 | 0/162 | 0.0 | --- | --- |
| TEA-Ch Map Mission, ss | Selective Attention | 7/56 | 12.5 | 1/130 | 0.8 | 2/155 | 1.3 | 21.61(<0.0001) | 18.95(<0.0001) |
| TMT – B, zs | Cognitive Flexibility | 51/56 | 91.1 | 100/137 | 73.0 | 101/160 | 63.1 | 16.14(<0.0001) | 13.58(0.001) |

**^(a)^** Two degrees of freedom.

**^(b)^** *P* values are exact 2-sided.

**^(c)^** Adjusted for the child’s sex, gestational age at birth, and small for gestational.

Statistically significant values of p<0.05 are shown in bold; p<0.004 meets significance based on Bonferroni correction

Note: All data presented is comparing attention classes based on performance on the cognitive measure of > 2 standard deviations below the mean.

Abbreviations for Measures: WMS – III, Wechsler Memory Scale – Third Edition; IVA, Integrated Visual and Auditory Continuous Performant Test; RCQ, Response Control Quotient; TEA-Ch, Test of Everyday Attention for Children, TMT – B, Trail Making Test, Part B; Stroop Interference, Stroop Color and Word Test, Interference Score; SS, Standard Score; ss, Scaled Score; zs, Z Score; SD, standard deviation; UA, Unaffected; SAL, School Age Limited; PIA, Persistent Inattentive.
